# RIPK3 Suppresses the Progression of Spontaneous Intestinal Tumorigenesis

**DOI:** 10.3389/fonc.2021.664927

**Published:** 2021-04-30

**Authors:** Qun Zhao, Jian Guo, Xinran Cheng, Yingying Liao, Yun Bi, Yingxia Gong, Xudong Zhang, Yang Guo, Xianhui Wang, Wei Yu, Shu Jin, Yan Tan, Xianjun Yu

**Affiliations:** ^1^ Laboratory of Inflammation and Molecular Pharmacology, Hubei Key Laboratory of Embryonic Stem Cell Research, School of Basic Medical Sciences & Biomedical Research Institute, Hubei University of Medicine, Shiyan, China; ^2^ Department of Gastroenterology, Renming Hospital, Hubei University of Medicine, Shiyan, China; ^3^ Oral Medicine Center, Renming Hospital, Hubei University of Medicine, Shiyan, China; ^4^ Department of Gastroenterology, Taihe Hospital, Hubei University of Medicine, Shiyan, China

**Keywords:** RIPK3, intestinal tumorigenesis, APC mutant mouse model, STAT3 (signal transducer and activator of transcription 3), IL-6 receptor antibody

## Abstract

Receptor-interacting protein 3 (RIPK3), a member of the family of serine/threonine protein kinases, emerged as a critical regulator of necroptosis. Downregulated expression of RIPK3 is correlated with poor prognosis in multiple tumor types. Here, we show that RIPK3 is involved in the progression of spontaneous intestinal tumorigenesis. As a clinical correlate, reduced expression of RIPK3 is positively associated with histological grade, lymphatic metastasis and poor prognosis in CRC patients. RIPK3-deficient (*Ripk3^-/-^*) mice exhibit increased tumor formation in *Apc^min/+^* spontaneous intestinal tumorigenesis. *Apc^min/+^Ripk3^-/-^* tumors promote hyperactivation of IL-6/STAT3 signaling, which exacerbates proliferation and inhibits apoptosis. Blocking IL-6 signaling suppressed tumor formation and reduced STAT3 activation in *Apc^min/+^Ripk3^-/-^* mice. Thus, our results reveal that RIPK3 is a tumor suppressor in spontaneous intestinal tumorigenesis, and implicate targeting the IL-6/STAT3 signaling axis as a potential therapeutic strategy for intestinal tumor patients with reduced RIPK3.

## Introduction

Colorectal cancer (CRC) is one of the most common cancers worldwide, and the number of deaths is rising in developing countries (1, 2). An increased frequency of CRC patients is usually diagnosed at an advance stage (3). Histologically, over 95% of CRC cases are adenocarcinomas, while squamous cell carcinomas, lymphomas and sarcomas are rarer (4). Increasing evidence reveals that excessive neoplastic transformation of colonic epithelial cells have the ability to initiate and develop CRC (5, 6). Moreover, CRCs arise in intestinal epithelial cells upon loss of tumor suppressor genes such as adenomatous polyposis coli (APC) (7). Thus, investigation of the molecular mechanisms in CRC tumorigenesis is urgently needed to prevent and treat CRC.

Inflammation is a dynamic and complex process to prevent overwhelming dampens and maintain tissue homeostasis (8, 9). Accumulation of the inflammatory environment drives intestinal cell proliferation and tissue regeneration through activation of pro-oncogene factors such as signal transducer and activator of transcription 3 (STAT3) (10, 11). Elevated levels of interleukin 6 (IL-6) are linked with chronic intestinal inflammation and tumorigenesis (12, 13). The role of IL-6/STAT3 signaling in intestinal cell proliferation and tumorigenesis is well established (14–16). A better understanding of the regulation of IL-6/STAT3 activation would facilitate the development of novel approaches for CRC therapy.

Necroptosis is a form of necrotic-regulated cell death upon extracellular and intracellular stimuli in certain pathologies (17–19). Receptor-interacting protein kinase 1 (RIPK1) and RIPK3 are critical regulatory molecules during necroptosis (20–22). RIPK1 interacts with RIPK3 *via* the RIP homotypic interaction motif (RHIM) domains and then forms the necrosome (23). RIPK1-RIPK3 necrosome complex results in the recruitment and phosphorylation of mixed-lineage kinase domain-like (MLKL), which leads to impair membrane integrity and drive necroptosis (24, 25). Several studies have shown that RIPK3 is involved in several inflammatory diseases, including intestinal inflammation, acute pancreatitis and skin inflammatory diseases (23, 26, 27). In fact, RIPK3 also exhibits a necroptosis-independent function in intestinal inflammation and tumorigenesis (28, 29). Reduced RIPK3 expression has been shown in human CRC tumors, and low expression of RIPK3 is significantly correlated with poor progression (30, 31). In contrast, overexpression of RIPK3 suppresses CRC cell proliferation, migration and invasion (32). In particular, loss of RIPK3 is susceptible to intestinal tumorigenesis in inflammation-associated colon cancer models (31, 33). However, colitis-associated tumorigenesis represents only 1% of colorectal cancer, and sporadic intestinal tumors are more prevalent (34). Thus, it is necessary to address the function of RIPK3 in the progression of spontaneous intestinal tumorigenesis.

In the present study, we revealed a tumor suppressor role for RIPK3 in spontaneous intestinal tumorigenesis. Expression analyses of RIPK3 expression showed that RIPK3 is significantly downregulated in CRC tumors, which predicted a poor prognosis in CRC. The absence of RIPK3 exhibits dramatically increased tumor numbers in *Apc^min/+^* mice through the hyperactivation of IL-6/STAT3 signals. Anti-IL-6R antibody therapy suppressed STAT3 activation and attenuated tumor burden in *Apc^min/+^Ripk3^-/-^* mice. Our findings highlight that reduced RIPK3 predicts a more aggressive disease and worse outcome in CRC.

## Materials and Methods

### Animal Experiments

All the mice were on a C57BL/6 background and maintained in a specific pathogen-free (SPF) facility. *Ripk3^-/-^* mice and *Apc^min/+^* mice have been described previously (29, 35). *Ripk3^-/-^* mice were crossed with *Apc^min/+^* mice to generate *Apc^min/+^Ripk3^+/-^* and *Ripk3^+/-^* mice. Then, *Apc^min/+^Ripk3^+/-^* mice were crossed with *Ripk3^+/-^* mice to generate *Apc^min/+^* and *Apc^min/+^Ripk3^-/-^* mice. This study was conducted according the guidelines of the Institutional Animal Care and Use Committee of the Hubei University of Medicine.

### Determination of Clinical Scores

Clinical scores were calculated as described in our previous studies (35). The disease activity index (DAI) was quantified with a clinical score assessment based on stool consistency and fecal blood. Briefly, stool scores were determined as follows: 0 = well-formed pellets; 1 = semi-formed stools in the anus and 2 = liquid stools adhered in the anus. The bleeding scores were determined as follows: 0 = no observed blood; 1= visible blood traces in stool; and 2= gross bleeding in rectal tissue.

### Tumor Load

Tumor load was determined according to a published protocol (36). Tumor load was calculated based on tumor numbers and tumor diameter.

### Tissue Homogenization and Western Blotting

Isolated intestinal tissues were homogenized using a Mini-Rotor (Thermo Scientific) and lysed in complete radioimmunoprecipitation assay (RIPA) buffer containing Roche’s cOmplete™ protease inhibitor cocktail. The protein concentrations were quantified by a quantitative BCA protein kit (P0010S; Beyotime Biotechnology, Shanghai, China). The proteins were separated by SDS-PAGE and detected using chemiluminescent substrate (Thermo Scientific). The following primary antibodies were used: rabbit anti-RIPK3 (#ab152130, Abcam; 1:1000), mouse anti-STAT3 (#9139, CST; 1:1000), rabbit anti-phospho-STAT3 (Tyr705) (#9145, CST; 1:1000), rabbit anti-PCNA (#13110, CST; 1:1000), rabbit anti-Cleaved-Caspase-3 (#9661, CST; 1:1000), and rabbit anti-Cyclin D1 (#2978, CST; 1:1000), and rabbit anti-C-myc (#9402, CST; 1:1000), and anti-GAPDH (#5174, CST; 1:5000).

### Quantitative Reverse-Transcriptase PCR (qRT-PCR)

Intestinal tissues were homogenized in TRIzol reagent (Life Technologies) to obtain RNA. RNA was reverse-transcribed to complementary DNA using HiScript III RT SuperMix for qPCR (gDNA wiper) (# R323, Vazyme Biotech Co.,Ltd), and the levels of genes were measured using quantitative RT-PCR using SYBR Premix Ex Taq™ (#RR420, TAKARA) according to the manufacturer’s instructions. mRNA quantities were normalized against that of GAPDH.

### Isolation of Colon Crypt Cells

Intestines were cleared and then cut into pieces. The colon crypt cells were collected following shaking after incubation in cold PBS with 3 mM EDTA/1.5 mM DTT.

### Intestinal Tissue for the Analysis of Cytokine Production

The intestinal tissues were collected and then homogenized in RIPA buffer containing Roche’s cOmplete™ protease inhibitor cocktail for 30 mins on ice. The protein concentration of IL-6 was measured by enzyme linked immunosorbent assay (ELISA) (eBioscience) (37).

### Anti-IL-6R Antibody Treatment

IL-6 signaling was antagonized with the IL-6R antibody tocilizumab (#A2012, Selleckchem; Houston, TX, USA). Four-week-old *Apc^min/+^Ripk3^-/-^* mice were intraperitoneally injected with 4 mg/kg tocilizumab weekly. After 10 weeks of treatment, all mice were euthanized, and the intestinal tissues were collected for further analysis.

### Statistical Analysis

The data are represented as the mean ± standard error of the mean (SEM) and were analyzed by GraphPad Prism software. Significant significance was determined by 2-tailed Student’s *t*-test, one-way ANOVA, Pearson’s correlation coefficients test, or log-rank test. *P* values below 0.05 were statistically significant.

## Results

### RIPK3 Expression Is Reduced in Colorectal Cancer (CRC) Patients

**Figure 1:**
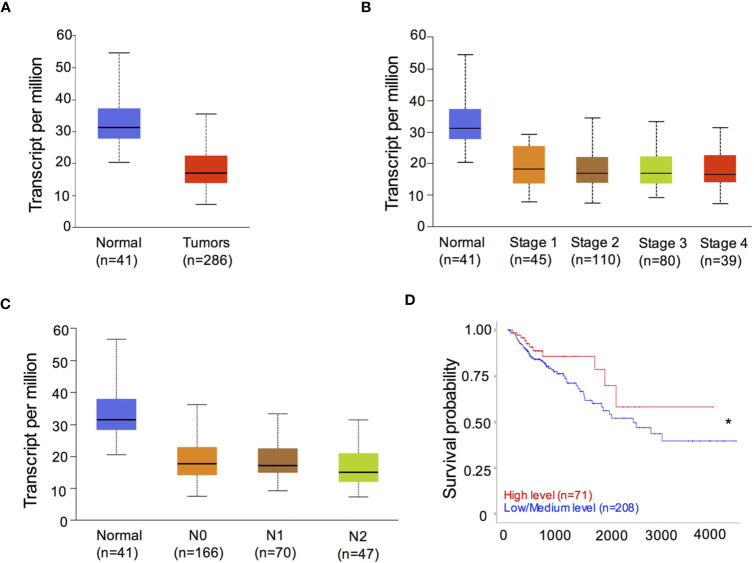
RIPK3 expression is reduced in colorectal cancer (CRC) patients. **(A)** The levels of *RIPK3* mRNA expression in colorectal cancer (CRC) and normal colorectal (NC) tissues in the TCGA colorectal database. n, sample numbers. **(B)** The levels of *RIPK3* mRNA expression in different stages of CRC progression. **(C)** The levels of *RIPK3* mRNA expression in metastatic tumors and normal counterparts. **(D)** Kaplan- Meier analyses of *RIPK3* expression in colorectal cancer patients. *p < 0.05.

To determine the role of RIPK3 in human CRC, we analyzed the levels of RIPK3 expression in containing 41 normal intestinal specimens and 286 CRC tumor samples from the Cancer Genome Atlas (TCGA) transcriptome database. CRC tumor samples showed significantly decreased expression of RIPK3 relative to normal intestinal tissue samples (**Figure 1A**). We next examined the levels of RIPK3 in different stages of CRC tumor progression. The results revealed that RIPK3 expression was higher in normal intestinal controls than in four CRC stages (**Figure 1B**). Interestingly, RIPK3 expression was lower in metastatic tumors than in normal counterparts (**Figure 1C**). More importantly, overall survival was significantly higher in patients with high RIPK3 expression than in patients with reduced RIPK3 expression in colorectal cancer (**Figure 1D**). These data indicate reduced RIPK3 expression in human CRC and suggest a suppressor role of RIPK3 in colorectal tumorigenesis.

### Loss of RIPK3 Aggravates Tumor Burden in *Apc^Min/+^* Mice

**Figure 2:**
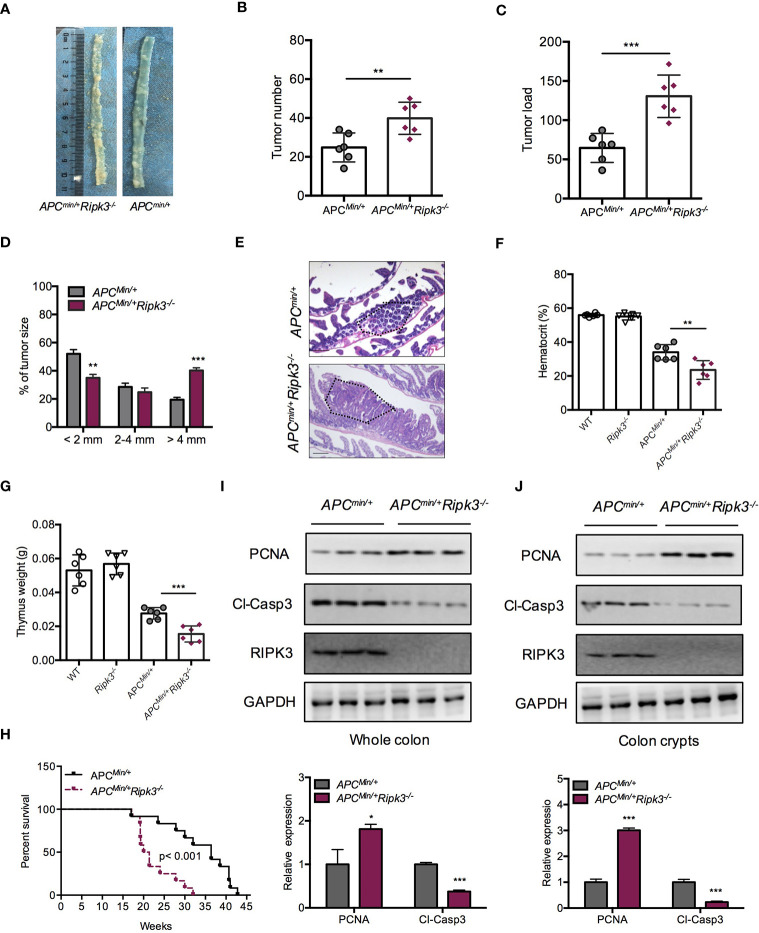
Loss of RIPK3 aggravates tumor burden in *Apc^Min/+^* mice. **(A)** Polyps in representative intestines from *Apc^min/+^* and *Apc^min/+^Ripk3^-/-^* mice. **(B, C)** Number of polyps formed **(B)** and tumor load **(C)** in 16-week-old *Apc^min/+^* and *Apc^min/+^Ripk3^-/-^* intestines. **(D)** Distribution of tumor size in 16-week-old *Apc^min/+^* and *Apc^min/+^Ripk3^-/-^* mice. **(E)** Images of the H&E-stained intestines from *Apc^min/+^* and *Apc^min/+^Ripk3^-/-^* mice. **(F, G)** Hematocrit **(F)** and thymus weight **(G)** of 16-week-old WT, *Ripk3^-/-^*, *Apc^min/+^* and *Apc^min/+^Ripk3^-/-^* mice. **(H)** Survival of *Apc^min/+^* and *Apc^min/+^Ripk3^-/-^* mice as indicated. *p < 0.05, **p < 0.01 and ***p < 0.001 versus *Apc^min/+^* mice. **(I, J)** Whole intestines and colonic crypts were isolated from 16-week-old *Apc^min/+^* and *Apc^min/+^Ripk3^-/-^* mice. PCNA and cleavage of caspase-3 were analyzed by western blotting. *p < 0.05 and ***p < 0.001 versus *Apc^min/+^* mice.

Mutations of the WT *Apc* allele (*Apc^min/+^*) are found in ~85% of intestinal tumors and promote intestinal tumorigenesis. *Apc^min/+^* mice are a suitable mouse model for the study of sporadic intestinal tumorigenesis. We hypothesized that RIPK3 deletion might increase the tumor burden in *Apc^min/+^* mice during intestinal tumorigenesis. To address the role of RIPK3 in intestinal tumorigenesis, *Apc^min/+^Ripk3^-/-^* mice were obtained by crossing *Apc^min/+^* mice with *Ripk3^-/-^* mice. Tumor progression in *Apc^min/+^Ripk3^-/-^* mice and those of *Apc^min/+^* mice was analyzed. The results showed significantly more lesions in the *Apc^min/+^Ripk3^-/-^* mice than in *Apc^min/+^* mice **(** **Figure 2A** **)**. Moreover, *Apc^min/+^Ripk3^-/-^* mice exhibited increased tumor numbers and tumor loads compared with in *Apc^min/+^* mice **(** **Figures 2B, C** **)**. Of note, the size of the tumor distribution in *Apc^min/+^Ripk3^-/-^* mice tended to be larger than *Apc^min/+^* mice **(** **Figure 2D** **)**. H&E staining also confirmed that there were larger polyps in *Apc^min/+^Ripk3^-/-^* intestines than in *Apc^min/+^* littermate controls **(** **Figure 2E** **)**. Accordingly, anemia and thymus atrophy were significantly exacerbated in *Apc^min/+^Ripk3^-/-^* mice compared to the *Apc^min/+^* littermate controls **(** **Figures 2F, G** **)**. Analyses of Kaplan-Meier survival found that the survival time of the *Apc^min/+^Ripk3^-/-^* mice was dramatically shorter, with a median survival of only 132 days relative to the median survival of the *Apc^min/+^* mice, which was 195 days **(** **Figure 2H** **)**.

Since loss of RIPK3 resulted in higher tumor burdens in the *Apc^min/+^* model, we hypothesized that RIPK3 inhibited tumor progression through regulating cellular proliferation. Compared with *Apc^min/+^* mice, *Apc^min/+^Ripk3^-/-^* tumors and colon crypt showed increased PCNA and decreased cleavage of caspase-3 proteins **(** **Figures 2I, J** **)**. However, there were no significant difference in proliferation and apoptosis was found in WT and *Ripk3^-/-^* intestines **(** **Figure S1** **)**, suggesting that loss of RIPK3 accelerates intestinal tumorigenesis by promoting proliferation and preventing apoptosis. These results demonstrate that loss of RIPK3 aggravates intestinal tumorigenesis in *Apc^min/+^* mice.

### Loss of RIPK3 Activates STAT3 Signaling Pathway in *Apc^min/+^* Mice

**Figure 3:**
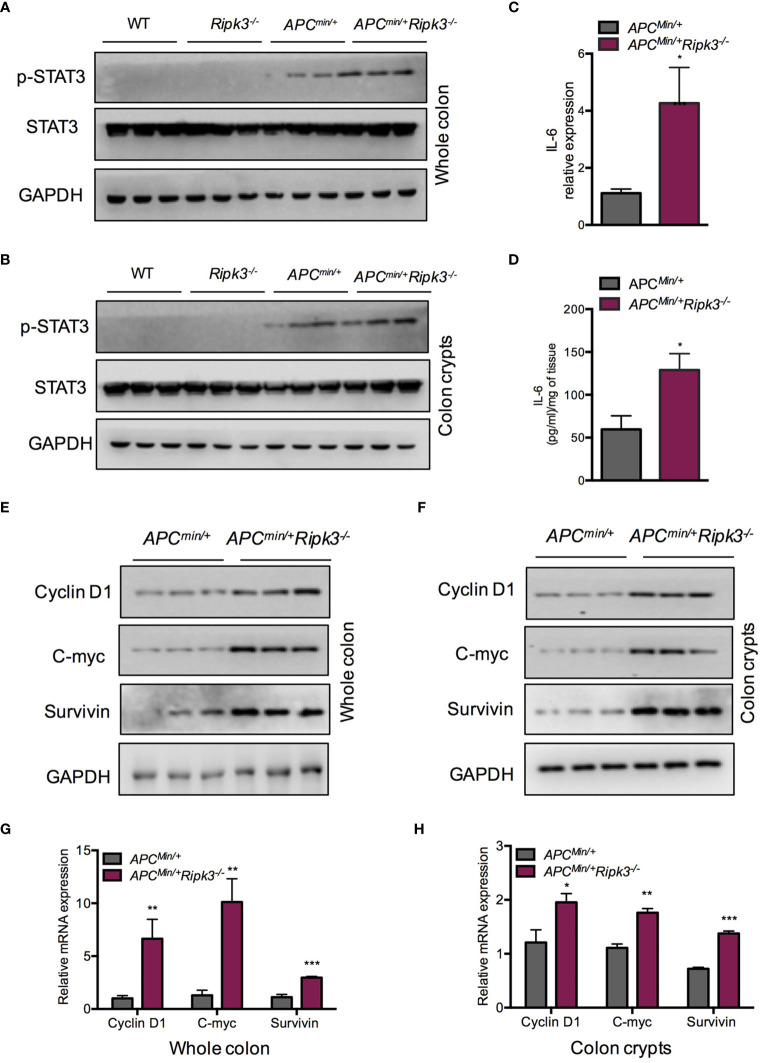
Loss of RIPK3 activates STAT3 signals in *Apc^min/+^* mice. **(A)** The expression of pSTAT3 in 6-week-old *Apc^min/+^* and *Apc^min/+^Ripk3^-/-^* intestinal tumors. **(B)** The levels of pSTAT3 in *Apc^min/+^* and *Apc^min/+^Ripk3^-/-^* colonic crypts. **(C)** Quantitative mRNA expression of IL-6 in *Apc^min/+^* and *Apc^min/+^Ripk3^-/-^* intestinal tumors. **(D)** The protein levels of IL-6 in *Apc^min/+^* and *Apc^min/+^Ripk3^-/-^* intestinal tumors as determined by ELISA. **(E)** The expression of STAT3 target genes in *Apc^min/+^* and *Apc^min/+^Ripk3^-/-^* intestinal tumors. **(F)** The expression of STAT3 target genes in *Apc^min/+^* and *Apc^min/+^Ripk3^-/-^* colonic crypts. **(G, H)** Quantitative mRNA expression of STAT3 target genes in *Apc^min/+^* and *Apc^min/+^Ripk3^-/-^* intestinal tumors and colonic crypts. *p < 0.05, **p < 0.01, ***p < 0.001 *versus Apc^min/+^* mice.

Activation of the STAT3 signaling pathway is associated with colorectal carcinogenesis and progression. In *Apc^min/+^* mice, hyperactivation of STAT3 accelerates intestinal tumorigenesis (38). To evaluate the status of STAT3 in intestinal epithelial cells and tumors from *Apc^min/+^Ripk3^-/-^* mice, we analyzed protein expression in intestinal tumors and colon crypt cells. The results indicated that the levels of pSTAT3 were higher in *Apc^min/+^Ripk3^-/-^* tumors than in *Apc^min/+^* tumors, WT and *Ripk3^-/-^* intestines (**Figure 3A**). We also found higher levels of pSTAT3 in *Apc^min/+^Ripk3^-/-^* colon crypts than in control mice (**Figure 3B**). As tumors from *Apc^min/+^Ripk3^-/-^* mice display activation of STAT3 signaling, we determined the reason for STAT3 hyperactivation in *Apc^min/+^Ripk3^-/-^* mice. IL-6 maintains persistent activation of STAT3 signaling in CRC (16). We found significantly increased levels of IL-6 mRNA and protein in *Apc^min/+^Ripk3^-/-^* tumors (**Figures 3C, D**). Aberrant activation of STAT3 promotes intestinal tumorigenesis by upregulating STAT3 target genes, which are involved in cell survival and proliferation (39). The results showed that the expression of STAT3 target genes (*Cyclin D1, C-myc* and *Survivin*) was increased in *Apc^min/+^Ripk3^-/-^* tumors compared with *Apc^min/+^* tumors (**Figure 3E**). Consistently, we found higher levels of these target genes in *Apc^min/+^Ripk3^-/-^* colon crypts than in *Apc^min/+^* mice (**Figure 3F**). Similarly, qRT-PCR results also showed significantly increased expression of these genes in *Apc^min/+^Ripk3^-/-^* tumors and colon crypts (**Figures 3G, H**). However, no remarkable increase in expression of STAT3 target genes was observed in either the intestines of the WT or *Ripk3^-/-^* intestines **(** **Figure S2**). These results indicate that IL-6/STAT3 signaling is activated in *Apc^min/+^Ripk3^-/-^* mice during intestinal tumorigenesis.

### Inhibition of IL-6 Blocks Tumor Burden in *Apc^min/+^Ripk3^-/-^* Mice

**Figure 4:**
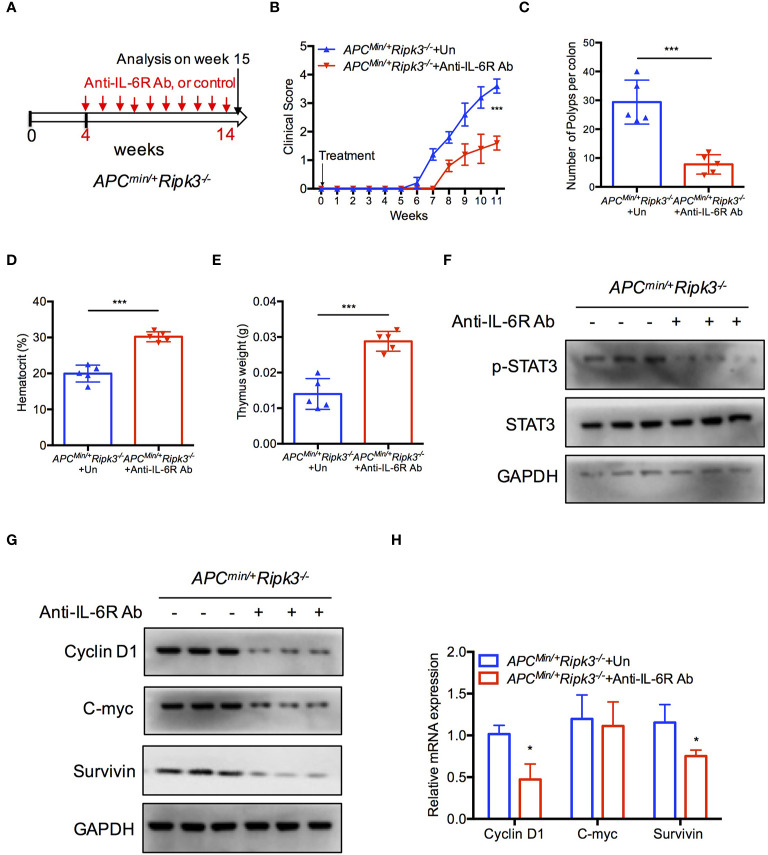
Inhibition of IL-6 blocks tumor burden in *Apc^min/+^Ripk3^-/-^* mice. **(A)** Scheme for anti-IL6R antibody treatment in *Apc^min/+^Ripk3^-/-^* mice. **(B)** Four-week-old *Apc^min/+^Ripk3^-/-^* mice were injected with anti-IL6R antibody with at a dosage of 4 mg/kg weekly for 10 weeks. The clinical scores were monitored. **(C)** Numbers of intestinal tumors in Un- or anti-IL6R-treated *Apc^min/+^Ripk3^-/-^* mice. **(D, E)** Hematocrit **(D)** and thymus weight **(E)** of *Apc^min/+^Ripk3^-/-^* mice after anti-IL6R therapy for 10 weeks. **(F)** Intestinal tumors were collected from PBS and anti-IL6R-treated *Apc^min/+^Ripk3^-/-^* mice. The levels of p-STAT3 were determined by western blotting. **(G, H)** The expression of STAT3 target genes expression in PBS and anti-IL6R-treated *Apc^min/+^Ripk3^-/-^* intestinal tumors. *p < 0.05, ***p < 0.001 *versus* Un-treated *Apc^min/+^Ripk3^-/-^* mice.

IL-6 is required for homeostasis of intestinal crypts (40, 41), and tumor formation is suppressed in the absence of IL-6 in the *Apc^min/+^* model (38, 42). To confirm the role of IL-6/STAT3 signaling in *Apc^min/+^Ripk3^-/-^* mice, we investigated the effect of IL-6 using an anti-IL-6 receptor (anti-IL-6R) antibody. We injected the anti-IL-6R antibody into *Apc^min/+^Ripk3^-/-^* mice for 10 weeks (**Figure 4A**). Treatment with anti-IL-6R antibody resulted in a lower clinical score in *Apc^min/+^Ripk3^-/-^* mice compared to the untreated (UT) mice (**Figure 4B**). After IL-6R antibody treatment for 10 weeks, the anti-IL6-R-treated *Apc^min/+^Ripk3^-/-^* mice showed fewer intestinal polyps than UT *Apc^min/+^Ripk3^-/-^* mice **(** **Figure 4C** **)**. As expected, anti-IL-6R antibody therapy alleviated anemia and thymus atrophy in *Apc^min/+^Ripk3^-/-^* mice **(** **Figures 4D, E** **)**. To elucidate whether STAT3 activation changes in anti-IL-6R-treated *Apc^min/+^Ripk3^-/-^* mice, intestinal polyps were isolated and analyzed by western blotting. Anti-IL-6R-treated *Apc^min/+^Ripk3^-/-^* intestines had decreased levels of p-STAT3 compared to UT *Apc^min/+^Ripk3^-/-^* mice **(** **Figure 4F** **)**. Consistently, the expression of STAT3 target genes Cyclin D1, C-myc and Survivin, was markedly suppressed in anti-IL-6R-treated *Apc^min/+^Ripk3^-/-^* tumors **(** **Figures 4G, H** **)**. These results demonstrate that increased IL-6/STAT3 signaling plays a causative role in the increased tumor burden in *Apc^min/+^Ripk3^-/-^* mice.

## Discussion

In this study, we found that loss of RIPK3 promoted the initiation and progression of spontaneous intestinal tumors. RIPK3 is reduced in human CRC patient cohorts and reduced RIPK3 is associated with poor prognosis in CRC. In the *Apc^min/+^* model, loss of RIPK3 elevated IL-6 levels, in turn hyperactivating the STAT3 signaling pathway and contributing to accelerated intestinal tumorigenesis. Notably, blocking IL-6 with a neutralizing IL-6 receptor antibody effectively attenuated tumor burden and STAT3 hyperactivation in *Apc^min/+^Ripk3^-/-^* mice. Our results highlight that RIPK3 plays a suppressive role in intestinal tumorigenesis by inactivating the IL-6/STAT3 signaling axis.

Necroptosis is involved in intestinal inflammation in mice and human IBD patients (43, 44). Abnormal RIPK3 expression is increased in Crohn’s disease (CD) and ulcerative colitis (UC) patients (44, 45). RIPK3 has a necroptosis-independent function by inhibiting inflammatory responses in DSS-induced acute colitis (28). In some types of tumors, reduced expression of RIPK3 appears to occur commonly and was found to correlate with reduced overall survival and poor prognosis (32, 46, 47). Previous studies have shown that RIPK3 suppresses inflammation-associated CRC (31, 33), while conflicting results suggest that RIPK3 promotes colitis-associated colorectal cancer (48). Recent work has examined the lack of an overt phenotype in *Ripk3^-/-^* mice in an inflammation-associated CRC model (49). These studies present conflicting results that might be due to differences in experimental conditions, commensal microflora and genetic background of mice. Thus, the results of a previous study indicate that the role of RIPK3 in the initiation and progression of CRC is still not clear. In addition, sporadic intestinal tumors are the majority of colon cancers, and the importance of RIRP3 needs to be addressed in sporadic intestinal tumors. Here, we took advantage of genetically engineered mouse models (GEMs) and established that RIRP3 deficiency drove increased tumor burden during intestinal tumorigenesis in *Apc^min/+^* mice. The present study revealed that the survival time was shorter in *Apc^min/+^* mice in the absence of RIRP3, which is consistent with reduced RIPK3 correlated with poor clinical outcomes in CRC. Consistently, we found reduced RIPK3 expression in human CRC compared with normal tissues using the TCGA database. RIPK3 expression was significantly decreased in four CRC stages versus in healthy controls. Importantly, significantly lower expression of RIPK3 predicted poor overall survival in CRC patients. In summary, these data reveal that RIPK3 exerts tumor-suppressive roles in intestinal tumorigenesis.

Intestinal tumors originate from epithelial cells through activation of multiple key signaling pathways for cell growth, differentiation, and survival. Compensatory proliferation and widespread apoptosis are known to contribute to promoting colorectal carcinogenesis. Here, our results indicated that *Apc^min/+^Ripk3^-/-^* tumors exhibited higher proliferative rates and reduced apoptotic compared to *Apc^min/+^* tumors. Notably, untransformed colon tissue and colon crypts of *Apc^min/+^Ripk3^-/-^* mice also exhibited higher proliferation and lower apoptosis. Thus, cooperation with increased proliferation and decreased apoptosis contributes to accelerated intestinal tumors in *Apc^min/+^Ripk3^-/-^* mice.

Inflammatory cytokines stimulate the activation of STAT3 signaling, which in turn contributes to promoted epithelial cell survival and resistance to apoptosis (5). STAT3 is critical for intestinal regeneration and tumorigenesis by regulating survival, cell cycle progression, and inflammation (50). Hyperactivation of STAT3 in epithelial cells has been linked to the development of CRC. Constitutively activated STAT3 in mice is resistant to intestinal tumor development (14). In contrast, IEC-specific STAT3-deficient mice exhibit decreased intestinal tumors (16). In this study, we observed that loss of RIPK3 promoted the activation of STAT3, thereby accelerating intestinal tumorigenesis in *Apc^min/+^* mice. Moreover, RIPK3-deficient mice exhibited increased expression of STAT3 target genes, including *Cyclin D1, C-myc* and *Survivin* in the *Apc^min/+^* model. Enhanced levels of IL-6 play a key role in STAT3 signaling in tumor formation and the development of CRC (42, 51, 52). Indeed, the levels of IL-6 were significantly upregulated in *Apc^min/+^Ripk3^-/-^* tumors. However, blocking IL-6 signals by anti-IL-6R antibody therapy was sufficient to suppress tumor formation in anti-IL6-R-treated *Apc^min/+^Ripk3^-/-^* mice. As expected, decreased levels of pSTAT3 and target genes were observed in anti-IL-6R-treated *Apc^min/+^Ripk3^-/-^* mice and correlated positively with decreased tumor numbers. Altogether, these data suggest that increased disease and polyp burden in *Apc^min/+^Ripk3^-/-^* mice are most likely mediated by hyperactivation of the IL-6/STAT3 signaling axis. The contribution of the cell-specific function of RIPK3 in the suppression of CRC needs to be further investigated using RIPK3 conditional knockout mice in future studies.

In summary, we report that RIPK3 exerts a suppressive role in intestinal tumorigenesis by suppressing the IL-6/STAT3 signaling axis. Mechanistic analysis provides evidence to further understand the role of RIPK3 in intestinal tumorigenesis and identify that increasing RIPK3 may be considered as a potential therapeutic strategy for CRC therapy.

## Data Availability Statement

The original contributions presented in the study are included in the article/**Supplementary Material**. Further inquiries can be directed to the corresponding authors.

## Ethics Statement

The animal study was reviewed and approved by Hubei University of Medicine. Written informed consent was obtained from the owners for the participation of their animals in this study.

## Author Contributions

XY and QZ conceived the study and designed the experiments. XY, JG, XC, YB, YG, XZ and QZ performed experiments. XY, JG, XC, YL, YG, XW, WY, SJ, YT and QZ analyzed data. XY and QZ wrote the manuscript. All authors contributed to the article and approved the submitted version.

## Funding

Our study was funded by the National Natural Science Foundation of China (81902852, 81502548), the Natural Science Foundation of Hubei Provincial Department of Education (D20202101), the Foundation of Health Commission of Hubei Province (WJ2019M053), the China Postdoctoral Science Foundation (2020M670220), the Biomedical Research Foundation, Hubei University of Medicine (HBMUPI201809), Faculty Development Grants from Hubei University of Medicine (2018QDJZR06), Open Project of Hubei Key Laboratory of Embryonic Stem Cell Research of Hubei University of Medicine (2020ESOF008), and Innovative Research Program for Graduates of Hubei University of Medicine (YC2020005), and the Student’s Platform for Innovation and Entrepreneurship Training Program (202010929001).

## Conflict of Interest

The authors declare that the research was conducted in the absence of any commercial or financial relationships that could be construed as a potential conflict of interest.
