# Supplementary material for: RIPK3 Suppresses the Progression of Spontaneous Intestinal Tumorigenesis
Source: Front Oncol. 2021 Apr 30;11:664927. doi: 10.3389/fonc.2021.664927 (PMC8120274; doi:10.3389/fonc.2021.664927)
Supplement: Supplementary file 1 [file DataSheet_1.docx]

**Appendix A. Supplementary material**

**Title: RIPK3 suppresses the progression of spontaneous intestinal tumorigenesis**

Qun Zhao ^1, #, *^, Jian Guo ^1, #^, Xinran Cheng ^1^, Yingying Liao ^2^, Yun Bi ^1^, Yingxia Gong ^1^, Xudong Zhang ^1^, Yang Guo ^1^, Xianhui Wang ^1^, Wei Yu ^3^, Shu Jin ^4^, Yan Tan ^1^, Xianjun Yu ^1,^ ^*^

**Supplemental Data**

**Figure S1, Related to Figure 2**

**Figure S2, Related to Figure 3**

**Supplemental figures and legends**


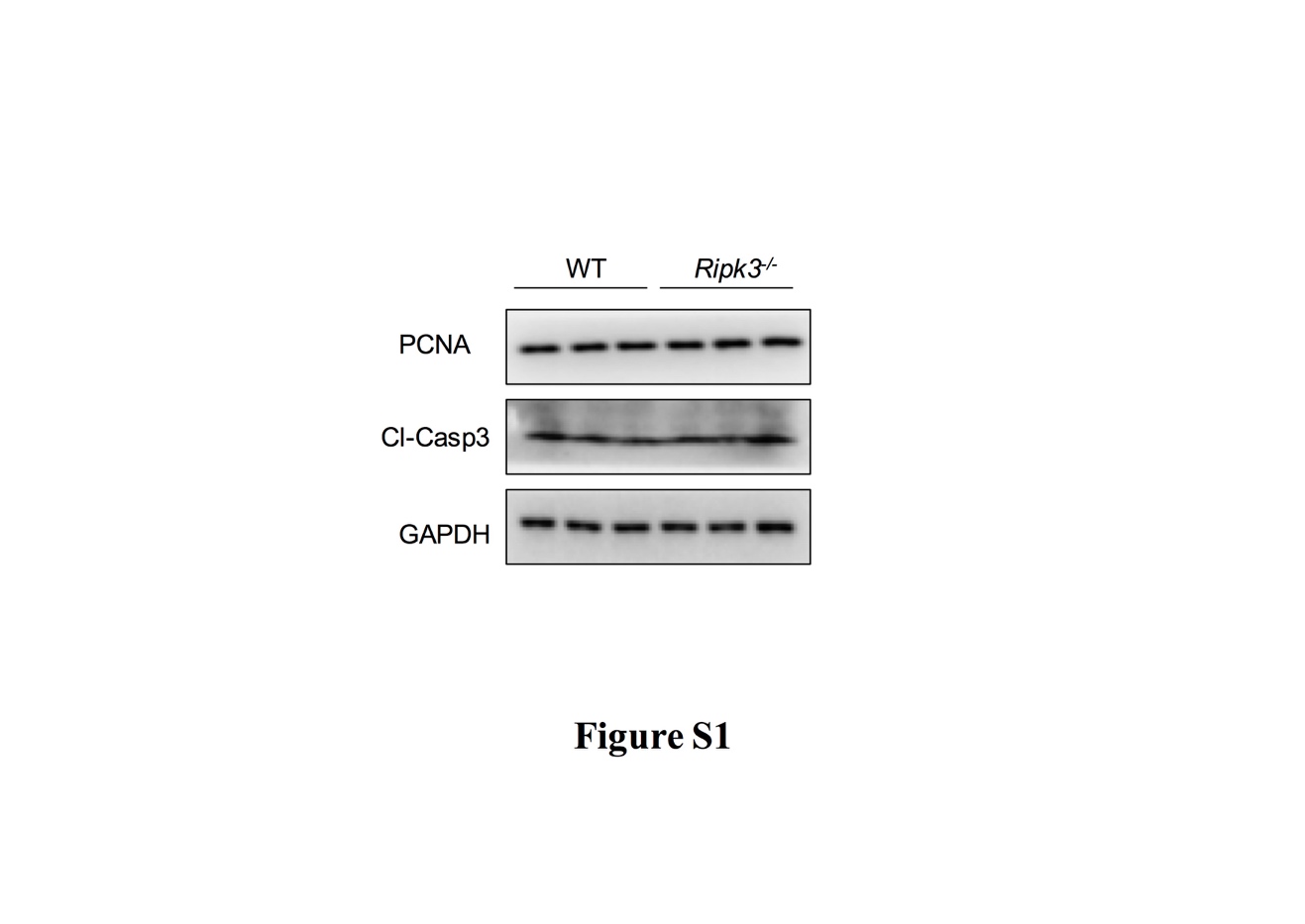


**Figure S1, Related to Figure 2. MLKL is dispensable for the proliferation and apoptosis under physiological conditions**

Whole intestines were isolated from 12-week-old WT and *Ripk3^-/-^* mice. PCNA and cleavage of caspase-3 were analyzed by western blotting.


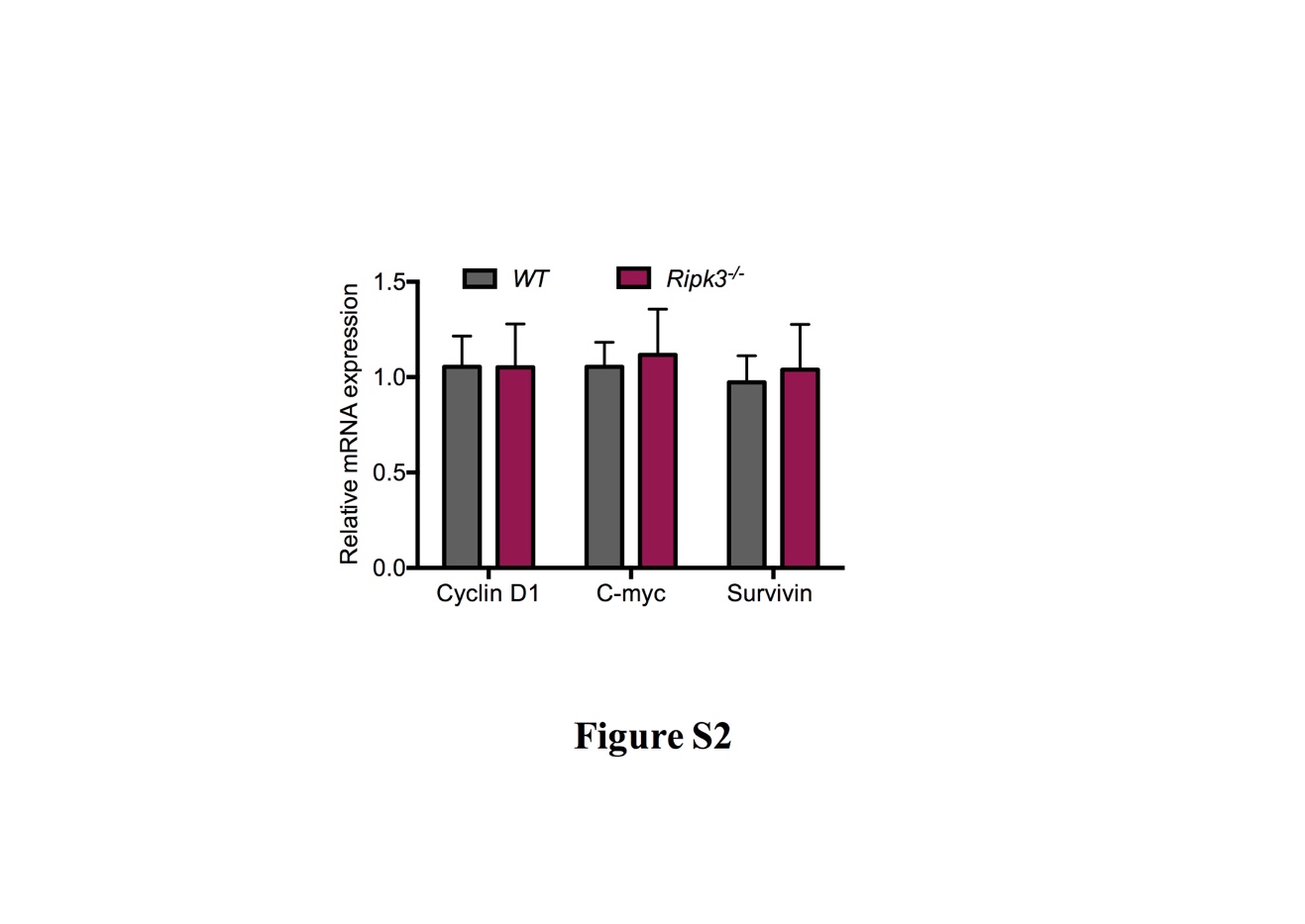


**Figure S2, Related to Figure 3. MLKL is dispensable for STAT3 activation under physiological conditions**

Whole intestines were isolated from 12-week-old WT and *Ripk3^-/-^* mice. Quantitative mRNA expression of STAT3 target genes in the intestines of WT and *Mlkl^-/-^* mice.
